# Etiopathological and hematobiochemical profiles in goats with gastrointestinal disorders

**DOI:** 10.14202/vetworld.2021.1760-1766

**Published:** 2021-07-06

**Authors:** Sunena Nayak, Prasana Kumar Rath, Susen Kumar Panda, Bidyut Prava Mishra, Rajshree Mishra, Shuvranshu Sekhar Biswal

**Affiliations:** 1 Department of Veterinary Pathology, College of Veterinary Science and Animal Husbandry, Odisha University of Agriculture and Technology, Bhubaneswar, Odisha, India; 2 Department of Livestock Products Technology, College of Veterinary Science and Animal Husbandry, Odisha University of Agriculture and Technology, Bhubaneswar, Odisha, India; 3 Department of Veterinary Microbiology, College of Veterinary Science and Animal Husbandry, Odisha University of Agriculture and Technology, Bhubaneswar, Odisha, India; 4 Teaching Veterinary Clinical Complex, College of Veterinary Science and Animal Husbandry, Odisha University of Agriculture and Technology, Bhubaneswar, Odisha, India

**Keywords:** diarrhea, goat, gastrointestinal disorders, hematobiochemical

## Abstract

**Background and Aim:**

Gastrointestinal (GI) disorders in small ruminants limit production efficiency and productivity growth in the livestock sector, thereby directly preventing farmers from augmenting their income. This study aimed to provide detailed insight into the etiology, hematobiochemical parameters, and epidemiological risk factors of GI disorders in goats and to determine the pathology associated with the disorders.

**Materials and Methods:**

Over the period of 2018-2019, 500 goats in and around Bhubaneswar, Odisha, India, were screened for GI disorders based on clinical signs. Blood samples from the control (n=10) and treatment (n=25) groups were collected for both hematological and serum biochemical alterations. Fecal examinations (n=220) were conducted for parasitic, bacterial, and virological assessments. Detailed necropsy and histopathological evaluations were conducted on 27 goats.

**Results:**

The GI disorder prevalence rate and mortality rate among the 500 goats analyzed were 44.4% and 12.27%, respectively. Chi-square analysis showed a significantly higher occurrence of GI disorders among the goats that were between 6 months and 1.5 years old (58.72%), were of the Ganjam breed (45.49%), had a poor body condition (71.11%), and were housed with an earthen floor (55.22%). The most common etiological risk factor observed was parasitic infection (65.45%), followed by bacterial (18.18%) and mixed infection (9.54%). Blood analysis showed neutrophilia and eosinophilia in infected goats, in addition to anemia; significant decreases in total protein, globulin, albumin, and glucose levels; and significant increases in aspartate transaminase and alanine aminotransferase levels. The major histopathological findings were infiltration of mononuclear cells and desquamation of the intestinal and ruminal mucosa.

**Conclusion:**

Stakeholders should focus not only on parasitic infections and other important etiological risk factors for GI disorders in goats but also on proper farming management practices to help enhance the income of farmers. The hematobiochemical alterations and pathomorphological changes reported in this study can be used by field veterinarians as guidelines for clinical evaluation and disease severity assessment.

## Introduction

Goats are considered to be one of the earliest domesticated animals and constitute 31% of the total livestock population, next to cattle, in Odisha, India (17.49°-22.34° N, 81.27°-87.29° E). Goat farming provides 25% of livelihood to Odisha’s rural population. India accounts for the largest livestock sector in the world, with goat farming contributing to 26.40% of the sector and thus playing a key role in the rural economy [1].

Gastrointestinal (GI) disorders are one of the important challenges affecting the profitability of goat husbandry practices in most countries, including India [2]. They lead to high mortality and morbidity rates, suboptimal productivity, increased sensitivity to diseases, banning of carcasses and organs, poor general health conditions, retarded growth, and high costs associated with preventive measures and veterinary aids [3]. The multifactorial etiology of GI disorders includes bacterial, viral, and parasitic infections and poor management practices [4]. The major clinical signs associated with GI disorders are diarrhea, anorexia, weight loss, abdominal distention, anemia, edema, general weakness, retarded growth, reduction in meat and milk yield, appetite loss, digestive inefficiency, severe debility, and even death in severe cases. Significant variations in hematobiochemical parameters have been observed in goats with GI disorders due to electrolyte loss [5].

Considering the socioeconomic impact of GI disorders in goats and the scarcity of related literature available, this study aimed to extensively characterize the epidemiology of hematobiochemical and pathological alterations in GI disorders, focusing on their complex etiology, in goats. It also aimed to educate and encourage livestock farmers and field veterinarians about taking suitable preventive and control measures to improve the efficiency of the production process and financial returns in the farming community.

## Materials and Methods

### Ethical approval

Goats presented to clinical complex of the college as well as local Veterinary dispensary were involved in the study. Blood samples were collected with due consent from animal owners by registered veterinarians for laboratory investigation. Adequate measures were taken to minimize pain or discomfort during the collection of clinical samples.

### <HStudy period and location

The study was conducted from June 2018 to June 2019 on the outskirts of Bhubaneswar, Odisha, India. The samples were processed at Department of Veterinary Pathology, College of Veterinary Science and Animal Husbandry, Odisha University of Agriculture and Technology, Bhubaneswar.

### 2>Screening of goats

A random screening of goats for GI disorders was conducted. Screening of goats was mostly relied on owners’ reports of their goats’ history and was based on the presence of clinical signs. Epidemiological risk factors, such as age, sex, breed, floor type, body condition, season, and possible involvement of etiological pathogens, were duly recorded during the study to determine their correlation with GI disorders in goats.

Blood samples from apparently healthy goats (control group, n=10) and goats with GI disorders (treatment group, n=25) were subjected to detailed hematobiochemical estimation. Possible etiological factors primarily involved in causing GI disorders were investigated through fecal sample examination and rectal swabs for any parasitic and bacteriologic involvement. Suspected cases were processed for the detection of viral etiology, with special reference to peste des petits ruminants (PPR). Goats that died during the study period (n=27) and showed signs of GI disorders, as per the history provided by their owners and data from the field veterinarians, were subjected to detailed necropsy and processed for routine histopathological hematoxylin-eosin staining, with special focus on the digestive organs, particularly the liver, intestine, and rumen, among others.

### Statistical analysis

Statistical analysis of all data recorded was performed using SAS software version 9.3, (SAS Institute Inc. Cary, NC, USA). Student’s *t*-test and Chi-square analysis were used to determine the significance of associations of the various risk factors with the occurrence of GI disorders in the goats.

## Results

The overall prevalence rate of GI disorders among the goats in this study was 44.4% (n=220); 27 goats died during the study period, giving an overall mortality rate of 12.27%. Mortality due to GI disorders as observed through necropsy was highest among kids <6 months old (51.85%), followed by goats between 6 months and 1.5 years old (33.34%), and lowest among adult goats >1.5 years old (14.81%). Breedwise, mortality was highest among the Ganjam breed (48.14%), followed by the Black Bengal breed (37.03%), and lowest among the indigenous/nondescript breeds (14.81%). Moreover, mortality in females (77.78%) was higher than that in males (22.22%).

**Figure-1:**
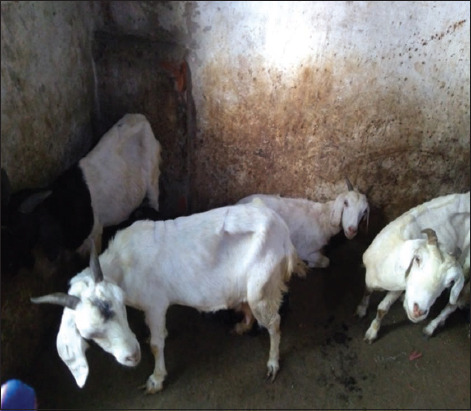
Reduced body weight and emaciation.

Details of the epidemiological risk factors associated with GI disorders are listed in Table-1. A higher prevalence of GI disorders was found among the 6 months-1.5 years age group (58.72%) and female goats (40%). The occurrence of GI disorders showed a non-significant association (p>0.05) with breed; relatively higher infections were observed in the Ganjam breed (45.49%), whereas the lowest occurrence was recorded for the Black Bengal breed (40%). Chi-square analysis revealed a significantly higher (p<0.05) prevalence of GI disorders during the rainy season (65%) and among goats with a poor body condition. This study also found a significantly higher (p<0.05) occurrence of GI disorders among goats housed with an earthen floor (55.22%) and the lowest occurrence in goats housed with a bamboo netting floor (28.41%). Furthermore, the clinical signs observed during the study were diarrhea, soiled hind quarter, anorexia, weakness, ruminal atony, tenesmus, edema, retarded growth and emaciation (Figure-1), recumbency, decreased body weight, appetite loss, digestive inefficiency, anemia, and debility.

**Table-1:** Epidemiological risk factors associated with gastrointestinal tract disorders in goats.

Risk factors	Groups	Healthy	Affected	%prevalence	Chi-square value	DF	p-value
Age	<6 months	103	67	39.41	41.97	2	<0.0001
	6 months-1.5 years	90	128	58.72			
	>1.5 years	87	25	22.32			
Sex	Male	50	20	4.00	7.86	1	<0.005
	Female	230	200	40.00			
Breed	Ganjam	133	111	22.20	0.71	2	<0.69
	Black Bengal	45	30	6.00			
	Indigenous	102	79	15.80			
Season	Rainy	70	130	65.00	59.87	2	<0.0001
	Summer	110	50	31.25			
	Winter	100	40	28.57			
Etiology	Parasite	-	144	65.45	500.00	4	<0.0001
	Bacteria	-	40	18.18			
	Mixed	-	21	09.54			
	None of the infections	-	15	06.81			
Body condition	Poor	65	160	71.11	143.28	2	<0.0001
	Moderate	75	45	37.50			
	Good	140	15	09.68			
Floor type	Cement	114	68	37.36	23.68	2	<0.0001
	Earthen	103	127	55.22			
	Bamboo netting	63	25	28.41			

Means having different superscripts in column are significantly different (p<0.05)

A significantly higher (p<0.05) occurrence of parasitic infection (65.45%) causing GI disorders was observed in this study, followed by bacterial (18.18%) and mixed infection (9.54%). None of the diarrheic goats screened positive for PPR infection based on molecular confirmation by polymerase chain reaction. Approximately 6.82% of the goats with GI disorders were not infected by infectious agents, such as bacteria and parasites. A total of 144 fecal samples tested positive for the presence of parasitic ova (Table-2). *Strongylus* spp. (39.88%) were the most prevalent bacteria detected followed by amphistomes (26.38%) and *Trichuris* spp. (14.58%). Fecal sample culture and Gram staining determined that *Escherichia coli* (67.5%) accounted for the highest proportion of bacterial etiology in 18.18% of the samples (n=40) followed by staphylococci (22.5%) and streptococci (10%).

**Table-2:** Prevalence of different parasites causing gastrointestinal tract disorders in goats.

Name of parasite	Type of parasite	No. of cases	Prevalence (%)
*Strongyle* spp.	Nematode	57	39.58
*Strongyloides* spp.	Nematode	9	6.25
Amphistome	Trematode	38	26.38
*Trichuris*	Nematode	21	14.58
*Coccidia*	Protozoa	13	9.02
*Fasciola*	Trematode	2	2.08
*Moneizia*	Cestode	2	2.08

Statistical analysis using Student’s t-test showed a significant difference (p<0.05) in various hematological parameters between the apparently healthy controls and the goats with GI disorders (Table-3). Neutrophilia and eosinophilia were detected in goats that screened positive for GI disorders. Anemia with decreased hemoglobin, total erythrocyte count, and packed cell volume values was also observed in infected goats. Some goats with GI disorders showed an increased total leukocyte count. Moreover, significant decreases (p<0.05) in globulin, albumin, total protein, and glucose levels and significant increases (p<0.05) in aspartate transaminase and alanine aminotransferase levels were seen in infected goats (Table-4).

**Table-3:** Mean±SE of hematological parameters.

Parameters	Control (n=10)	Affected (n=25)
Hb (g/dL)	11.56±0.12^a^	9.31±0.21^b^
TEC (M/µL)	15.41±0.04^a^	13.52±0.08^b^
PCV (%)	33.69±0.36^a^	27.86±0.63^b^
TLC (10^3^/µL)	10.57±0.03^a^	11.41±0.06^b^
MCV (fL)	21.85±0.24	20.58±0.42
MCH (pg)	7.50±0.08a	6.88±0.14^b^
MCHC (g/dL)	34.31±0.20^a^	33.46±0.34^b^
Neutrophil (%)	38.00±0.25^a^	43.64±0.87^b^
Lymphocyte (%)	56.30±0.26^a^	48.36±0.89^b^
Eosinophil (%)	4.00±0.21^a^	5.88±0.17^b^
Monocyte (%)	1.70±0.15^a^	2.12±0.12^b^

Means with different superscripts differ significantly (p<0.05). MCV=Mean corpuscular volume, MCH=Mean corpuscular hemoglobin, TLC=Total leucocyte count, Hb=Hemoglobin, MCV=Mean corpuscular volume

**Table-4:** Mean±SE of serum biochemical parameters.

Parameters	Control (n=10)	Affected (n=25)
Total protein (g/dL)	6.84±0.01^a^	6.52±0.01^b^
Glucose (mg/mL)	55.77±0.13^a^	53.41±0.09^b^
Albumin (g/dL)	3.13±0.02^a^	2.85±0.01^b^
Globulin (g/dL)	3.85±0.01^a^	3.66±0.01^b^
A:G ratio	0.81±0.00^a^	0.78±0.00^b^
AST (IU/L)	54.10±2.40^a^	75.76±0.90^b^
ALT (IU/L)	9.50±0.58^a^	18.20±0.43^b^

Means with different superscripts differ significantly (p<0.05). ALT=Alanine aminotransferase, AST=Aspartate aminotransferase

**Figure-2:**
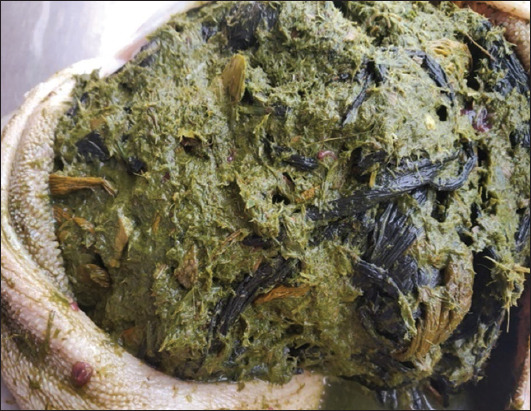
Plastic foreign body in the rumen.

Intestinal parasitism was the most prevalent pathological condition observed in this study, followed by pathomorphological infections of the liver and foreign body accumulation of plastic and polythene in the rumen (Figure-2 and Table-5). Enlargement of infected livers with rounded edges and a thickened capsule that had many hemorrhagic patches; congestion; necrotic foci; distention of the gallbladder with thick, sticky bile; and abscess was also seen. The spleen was congested in most of the carcasses. The major intestinal change noted was congestion with hemorrhagic and catarrhal enteritis. In few cases, mild-to-moderate thickening of the intestinal mucosa with scattered whitish plaques or nodules was observed. Most cases revealed amphistomes attached to the ruminal wall, which led to congestion, hemorrhage, erosive/ulcerative lesions, and sloughing of the ruminal mucosa and papilla. Plastic foreign bodies and polythene were found inside the rumen of five goat carcasses during necropsy.

**Table-5:** Gross pathological changes observed during necropsy.

Gross changes	Liver	Intestine	Rumen	Abomasum
Congestion	15	14	5	9
Hemorrhages	6	10	8	7
Necrotic foci	6	2	-	-
Catarrhal enteritis	-	12	-	-
Foreign body	-	-	05	-
gastrointestinal parasite	-	04	09	-

**Figure-3:**
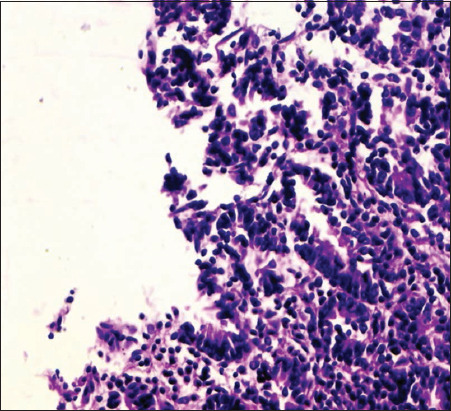
Photomicrograph showing desquamation of intestinal epithelium (H and E-40×).

**Figure-4:**
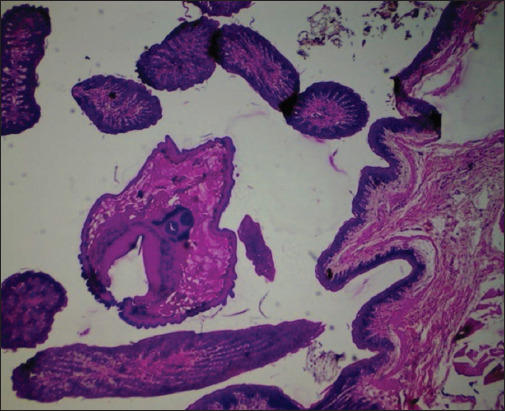
Photomicrograph showing cross-section of parasites in mucosal surface of rumen (H and E-4×).

**Figure-5:**
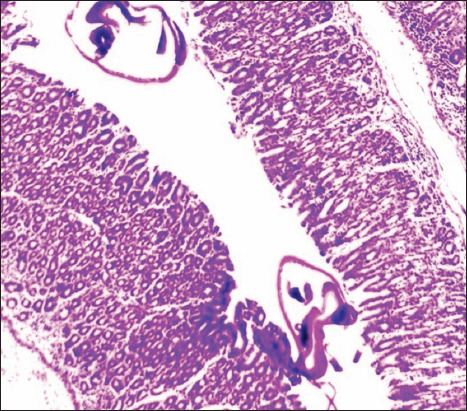
Photomicrograph showing cross-sections of parasite in mucosal fold of abomasum (H and E-10×).

Hemorrhages in hepatocytes along with centrilobular necrosis, infiltration of inflammatory cells with distended sinusoids, and hyperplasia of Kupffer cells were evident. Necrobiotic changes, such as cloudy swelling and fatty changes in hepatocytes, vacuolar degeneration of hepatocytes around the central vein with a vesicular nucleus and sinusoidal congestion, thickening of Glisson’s capsule with infiltration of inflammatory cells, and diffused fibrotic proliferation of the hepatic parenchyma, were observed. In few cases, bile duct hyperplasia with fibrotic proliferation was clearly present. Proliferation of goblet cells; infiltration of inflammatory cells, mostly lymphocytes, eosinophils, and macrophages; edema; congestion of the mucosa and submucosa; desquamation of the intestinal epithelium (Figure-3); hemorrhage; and villous atrophy with necrotic debris sticking to the mucosal surface of the intestine were common histological findings. Microscopic lesions of the rumen demonstrated hydropic degeneration, submucosal edema, disruption of the stratified epithelium with focal hyperplasia of the ruminal epithelium, and sloughing of the ruminal mucosa, thereby exposing the inner muscular layer. Leukocytic infiltrations in the mucosa and submucosa of the rumen and abomasum, in addition to congestion and, in some cases, cross-sections of parasites (Figures-4 and 5), were seen. Submucosal edema with infiltration of inflammatory cells and congestion of the reticular mucosa was also detected.

## Discussion

The prevalence and mortality patterns observed in goats with GI disorders in this study are in accordance with the findings reported by Islam *et al*. [6]. The significantly higher prevalence of GI disorders found among young growers might be due to their relatively higher exposure to risk factors from grazing contaminated pastures [7]. The females’ increased susceptibility to common pathogens is attributable to their genetic predisposition and varied hormonal status in different life stages, such as lactation and parturition [8]. The increased prevalence of GI disorders during the rainy season is related to the proliferation of pathogens in favorable wet environments. Poor body condition with suppressed body immune status might be an augmenting factor for more opportunistic infection, resulting in a higher prevalence of GI disorders [9]. By contrast, good body condition with an apparently healthy immune status definitely increases the body’s immune status and resistance to common infections, thereby lowering the risk of GI disorders in goats [6]. Use of bamboo netting over the floor reduces the probability of direct contamination with wet materials on the floor [10].

The clinical signs observed in this study are in agreement with those reported by Verma *et al*. [7]. Enterotoxins, such as Shiga toxins, produced by bacterial pathogens, particularly enterotoxigenic *E. coli*, cause damage to the intestinal mucosa, malabsorption due to the presence of GI helminths, intestinal obstruction due to the heavy load of GI parasites, and release of toxins from parasites, which impair liver function due to reduced digestive efficiency and diarrhea [11]. Tenesmus can be attributed to abdominal pain due to continuous irritation by GI parasites, whereas blood-sucking helminths cause anemia and edema. Intensive goat farming aiming for higher production and body growth might require feeding with more concentrates, which cause impaction and bloating, among others [12]. Diarrhea occurs due to enteric pathogens that mostly result in electrolyte loss and dehydration, which lead to recumbency and death [13]. Mechanical obstruction by immature and adult amphistomes in the rumen causes bloating, which leads to digestive disturbances [14].

None of the diarrheic goats in this study screened positive for PPR infection, which might be a result of the mass vaccination of goats against the PPR virus in the area. Ershaduzzaman *et al*. [15] reported simultaneous infection with GI helminths and bacteria that caused diarrhea in Bengal goats in Bangladesh. The high prevalence of parasitic infections causing GI disorders in goats may be due to their grazing in contaminated pastures and poor body condition, irregularity in timely deworming, suppressed immunity due to stress by lactation, and production of and association with various intermediate hosts, which make goats easy targets for parasitic infection [16]. The high prevalence of *Strongylus* worms might be due to the ability of parasite to direct life processes and to goats’ classic grazing behavior, which facilitates infection from contaminated pastures [17]. *E. coli* is considered a common intestinal microflora of warm-blooded animals that sometimes emerge as a pathogenic strain due to the transport-associated stress that these animals experience and their poor body condition and lower immune status, resulting in diarrhea [18].

The neutrophilia and eosinophilia observed in goats with GI disorders in this study were associated with parasitic and bacterial etiologies. Anemia could be related to the continuous blood loss triggered by GI helminths, mostly *Strongylus* spp., which are known to actively suck blood in the stomach and intestines [19]. The significant increases (p<0.05) in total and differential leukocyte count can be ascribed to bacterial and parasitic infections [13].

The serum biochemical profile observed in this study is in congruity with that reported by Pandit *et al*. [20]. Reduction in total protein and glucose levels in infected goats is due to inappetence, anorexia with reduced dietary protein intake, and malabsorption due to damage to the intestinal mucosa by toxins released by intestinal pathogens, leading to poor absorption of protein metabolites [13]. Continuous blood sucking by GI helminths also results in loss of plasma proteins, leading to hypoproteinemia and ascites. Toxins released by parasites and bacteria could damage the liver, thereby reducing protein synthesis [13]. Living parasites might consume glucose, consequently reducing the serum glucose concentration in infected goats [14]. Hepatic damage associated with parasitic and bacterial infections may suppress the metabolic activity of the liver, thus decreasing albumin and globulin concentrations in infected goats. Significant increases in aspartate transaminase and alanine aminotransferase levels can be attributed to severe damage to hepatocytes and intestines associated with bacterial and parasitic infections [21].

The pathomorphological alterations observed in goats infected with GI disorders in this study are in agreement with those reported by Al-Qudah *et al*. [12]. Most gross lesions are associated with bacterial toxins that lead to acute to chronic inflammatory reactions, which result in congestion and hemorrhage [13]. Mechanical irritations during the migration of GI helminths and parasitic toxins produce characteristic morphometric alterations in different organs [22]. Foreign bodies in the rumen [23] might be due to the development of pica, which is associated with nutritional deficiency and poor management practices [24,25]. The histopathological lesions observed in different organs of the goats in this study suggest degenerative and inflammatory responses and are in general agreement with those reported by Tariq *et al*. [25] and Hajimohammadi *et al*. [26].

## Conclusion

This study found a higher occurrence of GI disorders that were significantly associated with various epidemiological risk factors in goats. The findings indicate the need to adopt improved hygiene and better management practices to curb GI infections with a complex multifactorial etiology suggestive of parasitic and bacteriologic involvement. Significant alterations in hematobiochemical parameters that corroborate gross and microscopic lesions, which contribute to suboptimal productivity in ruminant livestock, should be addressed properly and urgently by all stakeholders to ensure production efficiency and productivity growth and thus help enhance the income of farmers.

## Authors’ Contributions

SN: Performed the field works pertaining to collection of the samples and carried out the laboratory work. PKR and SKP: Designed the work and wrote the manuscript. BPM, RM, and SSB: Revised the manuscript, collected the scientific literature, and helped in statistical analysis. All authors read and approved the final manuscript.
